# Editorial

**Published:** 1873-06-15

**Authors:** 


### MEDICAL EXAMINER.

*A Semi-Monthly Journal of Medical Sciences.*

EDITED BY

N. S. DAVIS, M. D., AND F. H. DAVIS, M. D.

Chicago, June 15th, 1873.

### EDITORIAL.

INDIANA STATE MEDICAL SOCIETY.—The annual meeting of this Society was recently held in Indianapolis, and appears to have been well attended. A goodly number of reports and papers, on topics of professional interest, were presented and discussed. Some measures were taken for increasing the number and efficiency of the County Medical Societies, and for influencing the Legislature in favor of a law legalizing the study of human anatomy by dissections.

The following officers were elected for the ensuing year: President, I. Casselberry, M.D., of Evansville; Vice-President, W. Hobbs, M.D., of Carthage; Secretary, G. V. Woolen, M.D., of Indianapolis; Assistant Secretary, W. J. Elstun, M.D., of Indianapolis; Treasurer, J. H. Woodburn, M.D., of Indianapolis; Librarian, A. W. Davis, M.D., of Indianapolis.

The business of the Society occupied two days, and was followed by a pleasant social entertainment on the evening of the second day.

IOWA STATE MEDICAL SOCIETY.—This Society held its annual meeting in Marshalltown, May 29, 1873. The time of the meeting was fully and profitably occupied in the hearing and discussion of reports and papers on medical subjects. Much attention was given to the history of diseases in the several counties during the past year. Dr. T. D. Fitch, of this city, was present as delegate from the Illinois State Medical Society. The following are the officers chosen for the ensuing year: President, W. S. Robertson, M.D., of Muscatine; 1st Vice-President, Wm. Carnes, M.D., of Iowa City; 2d Vice-President, John Bower, M.D., of Guthrie Centre; Secretary, S. B. Thorn, M.D., of Otumwa; Assistant Secretary, J. R. Kennedy, M.D., of DesMoines; Treasurer, J. W. Gustin, M.D., of Burlington.

NATIONAL MEDICAL REGISTER.—We learn from Dr. S. W. Butler, of Philadelphia, who has this work in press, that he has sent his circulars to the physicians of the Northwestern States, and is anxiously waiting for replies. This Medical Register is a very important work, and it is equally important that the name of every regular physician should appear in it *correctly*. Hence, all who have received circulars asking for information from Dr. Butler, should return them with the proper answers without delay.

ROCK RIVER MEDICAL SOCIETY.—The annual meeting of this Society was held in Theresa, Wis., May 14th, 1873. The following officers were elected for the ensuing year: President, Dr. L. Loehr, Theresa; Vice-President, Dr. A. W. Lueck, Mayville; Secretary and Treasurer, Dr. N. Senn, Ashford; Board of Censors, Drs. Senn, Marston and Rogers. The next meeting will be held in Theresa, at the Rock River House, on the second Wednesday in July next.

THE OHIO STATE MEDICAL SOCIETY held its annual session in Columbus, June 9th and 10th. Toledo was selected as the next place of meeting, and the following officers were elected for the ensuing year: H. J. Harrick, of Alvond, President; M. J. Conklin, of Dayton, Secretary; H. Edwards, of Lancaster, Assistant Secretary; and S. S. Gray, of Piqua, Librarian and Treasurer.

THE UPPER CEDAR VALLEY MEDICAL ASSOCIATION held its annual meeting June 10th, 1873, at Charles City, Iowa. The officers elected for the ensuing year were, President, Dr. J. W. Smith, Charles City; Vice-President, Dr. Noyes, Mason City; Secretary, Dr. L. P. Fitch, Charles City; Treasurer, Dr. W. F. Pitts, Frederickburg.

